# Melatonin and mitochondrial protection in cardiac ischemia–reperfusion injury: mechanisms, evidence and translational perspectives

**DOI:** 10.1007/s00395-026-01162-z

**Published:** 2026-02-24

**Authors:** Gaia Pedriali, Sara Leo, Margherita Tiezzi, Elena Nicoletta Colarusso, Giampaolo Morciano, Elena Tremoli, Paolo Pinton

**Affiliations:** 1 Maria Cecilia Hospital, GVM Care & Research, Cotignola, Italy; 2 Department of Medical Sciences, Section of Experimental Medicine and Technopole of Ferrara, Laboratory for Advanced Therapies (LTTA), University of Ferrara, Ferrara, Italy; 3 Department of Biosciences, Biotechnologies and Environment, University of Bari “A. Moro”, Bari, Italy

**Keywords:** Mitochondria, Cardiac ischemia reperfusion injury, Melatonin, Cardioprotection

## Abstract

Cardiac ischemia–reperfusion injury (IRI) leads to significant mitochondrial impairment, which contributes to cell death and hampers myocardial recovery. During IRI, mitochondria are subjected to oxidative stress, calcium overload, and altered dynamics, resulting in the opening of the mitochondrial permeability transition pore (mPTP), release of cytochrome c, and activation of apoptotic pathways. Melatonin, a pleiotropic indoleamine produced by the pineal gland and other tissues, has cardioprotective effects through both direct antioxidant activity and receptor-mediated mechanisms. This review explores melatonin’s role in maintaining mitochondrial integrity under IRI conditions. Melatonin counteracts oxidative damage by neutralizing reactive oxygen species, stabilizing mitochondrial membrane potential, and preventing mPTP opening, thereby reducing activation of cell death pathways. It also supports mitochondrial biogenesis and dynamics, contributing to energy balance and reduced oxidative burden. In addition, melatonin regulates mitophagy, ensuring mitochondrial quality control and preventing excessive degradation, which collectively contributes to restoring mitochondrial function and cellular metabolism. In rodent preclinical models, melatonin administration before ischemia, during ischemia, or at reperfusion has consistently reduced infarct size and improved cardiac function. While these preclinical findings are encouraging, studies on rabbits or pigs and clinical studies have not consistently replicated these benefits. The variability in outcomes may be attributed to differences in study design, timing and method of melatonin administration, and types of endpoints measured. Comorbidities, risk factors, and comedications further influence mitochondrial biology and melatonin’s efficacy in cardiac IRI. A dedicated comparative analysis evaluates melatonin against established and emerging cardioprotective approaches targeting mitochondria, underscoring its potential for combination therapies.

## Introduction

Cardiac ischemia–reperfusion injury (IRI) is a pathological condition that occurs after restoration of blood flow to ischemic tissue, characterized by a worsening of cellular functions and tissue damage. The interruption of blood flow and subsequent reoxygenation trigger a series of complex biochemical and cellular events that exacerbate myocardial injury. While timely reperfusion is mandatory to restore oxygen and nutrients to the affected myocardium, it paradoxically leads to further damage through oxidative stress and mitochondrial dysfunction [178]. Given that IRI is a common mechanism underlying several diseases (such as myocardial infarction (MI), stroke, and major surgeries [48, 75]), extensive research focused on the complexity of molecular pathways involved with the aim of improving clinical interventions and prognosis, as well as developing new therapeutic strategies.

Mitochondria play a crucial role in the cardiac IRI process due to their significance in energy supply and metabolism [81]. During ischemia, ATP synthesis becomes compromised and intracellular calcium (Ca^2+^) homeostasis is disrupted. Although proper mitochondrial Ca^2^⁺ handling is essential for maintaining energy homeostasis and cardioprotection, Ca^2^⁺ overload represents a critical trigger of irreversible mitochondrial injury and cardiomyocyte death [16]. Upon reperfusion, mitochondria buffer Ca^2^⁺, oxidative stress is augmented; this promotes the opening of the mitochondrial permeability transition pore (mPTP) [19] and ultimately the induction of cell death [105, 178]. The mPTP is a non-selective channel that opens in response to excessive mitochondrial reactive oxygen species (ROS) and Ca^2+^ overload. Its opening compromises IMM integrity and is a critical event leading to the release of pro-apoptotic factors. Under normal conditions, the IMM is highly impermeable to most ions and metabolites; however, during myocardial IRI, pathological and transient mPTP opening causes mitochondrial swelling and membrane rupture, allowing cytochrome c to cross the outer mitochondrial membrane (OMM) and initiate cell death signaling pathways [21, 231]. In addition to Ca^2^⁺ overload and oxidative stress, alterations in mitochondrial membrane composition have emerged as important determinants of reperfusion injury [30]. In fact, it has been demonstrated that ischemia–reperfusion (I/R) induces profound remodeling of the cardiac lipidome at the cellular level, highlighting membrane vulnerability to oxidative damage and underscoring the central role of lipid alterations in myocardial injury [158].

During the last years, several interesting approaches have been explored to target mitochondrial function during this process, including mPTP opening inhibitors, molecules regulating mitochondrial dynamics, mitophagy inducers, and antioxidant drugs [29, 51, 151, 163]. Among these, melatonin has been considered due to its ability to modulate mitochondrial activity. Melatonin is an endogenous hormone synthesized by the pineal gland with the main role of controlling circadian rhythm. It is a highly lipophilic and hydrophilic molecule that can cross cellular membranes and reach cytoplasm and nucleus [118]. Melatonin exerts broad protective effects on mitochondria, even under ischemic stress, by scavenging ROS, enhancing antioxidant enzyme activity, preserving electron transport chain function and ATP production, and maintaining mitochondrial structural integrity, including cardiolipin and mitochondrial mass [91]. Experimental evidence of the cardioprotective effects of melatonin against IRI has been gathered for at least 30 years [119, 198, 218]. For example, a meta-analysis of 11 preclinical studies concluded that melatonin pretreatment confers significant cardioprotection, as evidenced by a reduction in infarct size and improvement in cardiac function [150]. However, the promising results observed in these preclinical studies have not been replicated in other animal models of IRI (rabbits, pigs) or in clinical trials, resulting in neutral outcomes regarding cardioprotection (Tables 1 and 2). The final examination of the main factors contributing to the discrepancy between preclinical and clinical findings for mitochondria-targeted cardioprotective compounds explores potential strategies to address these challenges aiming to identify approaches to improve translational success.

**Table 1:** Preclinical studies evaluating melatonin as a therapeutic strategy against cardiac IRI

Reference	Model	Treatment	Results
[77]	Isolated perfused rat hearts subjected to regional I/R	Melatonin 50 µM administered before and after ischemia. Luzindole 5 µM	Melatonin reduced infarct size via a melatonin receptor-dependent mechanism
[246]	In vivo Sprague Dawley rats subjected to myocardial I/R	Melatonin 10 mg/kg/day for 4 weeks	Melatonin improved cardiac function, reduced apoptosis and oxidative damage in a melatonin receptor-dependent manner
[90]	Mice (wild-type, MT1- or MT2-silenced, MT1- or MT2-overexpressing) subjected to myocardial IRI	Melatonin 20 mg/kg + genetic modulation of MT1/MT2	Melatonin reduced infarct size, oxidative/nitrative stress, ER stress, mitochondrial injury, and apoptosis via activation of MT2 (but not MT1). Overexpression of MT2 protected, whereas MT2 silencing abolished the benefit
[205]	Male Wistar rats subjected to in vivo cardiac I/R with left anterior descending artery (LAD) ligation	Melatonin 10 mg/kg given as pretreatment, during ischemia, or at reperfusion + receptor blockers (Luzindole 1 mg/kg; 4-PPDOT 1 mg/kg)	Melatonin reduced infarct size, arrhythmias, and LV dysfunction regardless of timing; preserved mitochondrial function, balanced dynamics, reduced excessive mitophagy, and decreased apoptosis via MT2 activation
[210]	Isolated Wistar rat hearts (Langendorff) subjected to 33 min global ischemia + 60 min reperfusion	Ramelteon (0.01–5 µM) perfused before ischemia; plus co-treatment luzindole 5 µM	Ramelteon (0.03 µM) significantly reduced infarct size. Cardioprotection was blocked by luzindole, demonstrating that ramelteon’s effect depends on melatonin receptors
[211]	Isolated Langendorff-perfused hearts from male Wistar rats (33 min global ischemia, 60 min reperfusion)	Ramelteon (0.03 µM) ± MT2 antagonist (4P-PDOT 1 µM)	Ramelteon significantly reduced infarct size, and this effect was abolished by MT2 inhibition
[107]	Aged (22–24 months) Wistar rat hearts on a Langendorff apparatus after 30 min LAD ligation + 60 min reperfusion	Melatonin 50 µM added to perfusion solution 5 min before reperfusion and during first 15 min of reperfusion	Melatonin improved hemodynamic parameters, reduced infarct size, decreased mitochondrial ROS, stabilized mitochondrial membrane potential
[239]	Isolated Langendorff-perfused rat hearts	Melatonin 5 µM pretreatment before I/R	Melatonin improved post-ischemic heart function, reduced infarct size, apoptosis, LDH release, restored mitochondrial redox potential, increased mitochondrial SOD, and reduced mitochondrial H₂O₂ and MDA via the activation of the JAK2/STAT3 signaling pathway
[140]	In vivo Wistar rats subjected to coronary artery occlusion (ischemia) followed by reperfusion	Melatonin (2.5, 5 and 10 mg/kg, administered intraperitoneally before ischemia)	Melatonin reduced infarct size, improved hemodynamic recovery, lowered serum markers of damage (CK-MB) and decreased oxidative stress
[206]	Prediabetic obese (high-fat diet) Wistar rats subjected to 30 min myocardial ischemia + 120 min reperfusion	Single-dose melatonin (10 mg/kg pretreatment or 20 mg/kg during ischemia or at reperfusion) ± Luzindole or 4-PPDOT 1 mg/kg	Melatonin (10 mg/kg before ischemia) reduced infarct size, arrhythmias, and LV dysfunction; improved mitochondrial function, reduced oxidative stress, restored the balance of mitochondrial dynamics, autophagy and apoptosis. Higher dose (20 mg/kg) was required when given during ischemia or at reperfusion. Protection was abrogated by MT2 blocker
[181]	Isolated Langendorff-perfused rat hearts subjected to global I/R	Melatonin 50 μM	Melatonin improved functional recovery, reduced infarct size, reduced LDH release; isolated mitochondria from treated hearts were more resistant to Ca^2^⁺‑induced mPTP opening, and melatonin prevented cytochrome c release as well as cardiolipin oxidation
[182]	Isolated heart mitochondria from aged (24 months) and young (5 months) rats	Long-term melatonin (≈ 10 mg/kg/day for 2 months) administered in vivo	In aged rat mitochondria, melatonin reduced susceptibility to Ca^2^⁺-induced mPTP opening, reduced cytochrome c release, and prevented cardiolipin oxidation, thereby improving mitochondrial stability
[253]	In vivo mice with LAD ligation 30 min cardiac I/R + 2 h reperfusion and isolated cardiac microvascular endothelial cells (CMECs)	Melatonin 20 mg/kg intraperitoneally 12 h before IRI	Melatonin reduced infarct size, preserved microvascular perfusion, maintained endothelial barrier integrity, reduced inflammation and CMEC death. Melatonin inhibited Drp1-dependent mitochondrial fission, prevented VDAC1 oligomerization and HK2 dissociation, suppressed mPTP opening, and blocked PINK1/Parkin-mediated mitophagy
[245]	In vivo type 1 diabetic rats subjected to myocardial ischemia/reperfusion injury with LAD ligation	Melatonin (dose: 10 mg/kg, intraperitoneal) before I/R	Melatonin significantly reduced infarct size, improved cardiac function, preserved mitochondrial bioenergetics, enhanced mitochondrial biogenesis, and maintained mitochondrial integrity via activation of the AMPK–PGC1α–SIRT3 signaling pathway
[230]	Male Sprague–Dawley rats subjected to myocardial I/R injury	Melatonin given 10 min before reperfusion 20–60 mg/kg	Melatonin reduced infarct size, improved mitochondrial function, lowered oxidative stress and suppressed excessive mitophagy; inhibition of SIRT3 abolished protection
[57]	Isolated perfused Wistar rat hearts subjected to 20 min global ischemia + 30 min reperfusion	Melatonin (0.3 µM or 50 µM) administered 10 min before ischemia and 10 min at reperfusion	Melatonin attenuated I/R-induced alterations in mitochondrial dynamics and quality control: reduced mitochondrial fission, modulated mitophagy markers, and prevented excessive mitophagy activation, promoting cardioprotection
[251]	In vivo mice with LAD ligation 30 min cardiac I/R + 2 h reperfusion and platelets from rats exposed to I/R conditions	Melatonin 20 mg/kg, intraperitoneal administration before I/R	Melatonin significantly suppressed platelet activation and aggregation, preserved platelet mitochondrial integrity, restored platelet PPARγ levels, inhibited FUNDC1-dependent mitophagy, and reduced I/R-induced myocardial injury
[249]	In vivo cardiac-specific KO mice with lLAD) ligation	Melatonin 20 mg/kg before I/R/during reperfusion	Melatonin preserved cardiac function and cardiomyocyte viability, restored mitochondrial fusion and mitophagy via upregulation of OPA1, normalized mitochondrial dynamics, enhanced mitochondrial energy metabolism; effects required the AMPK signaling pathway
[146]	In vivo mice with LAD ligation	Melatonin 10 and 20 mg/kg administered before I/R	Melatonin reduced IRI, promoted mitochondrial fusion via upregulation of OPA1, through activation of the YAP–Hippo pathway
[62]	Closed-chest porcine model of myocardial I/R	Melatonin, 200 mg total dose (0.4 mg/mL), administered intracoronary and intravenous at reperfusion	Melatonin did not significantly improve myocardial salvage index, did not reduce microvascular obstruction, and only non-significantly reduced troponin T release
[88]	Closed-chest porcine model of myocardial I/R	Intravenous and intracoronary melatonin, total dose 200 mg (0.4 mg/mL) administered immediately prior to reperfusion	Melatonin did not reduce circulating oxidative (MDA) or inflammatory markers (IL-1β, IL-6, IL-10), nor high-sensitivity troponin T
[46]	Male New Zealand rabbits subjected to 30 min coronary artery occlusion and 3 h reperfusion	Melatonin 10 and 50 mg/kg intravenous administration 10 min before occlusion + 15 min before reperfusion	Melatonin had no significant effect on hemodynamics (heart rate, blood pressure), coronary blood flow, or infarct size compared to control
[5]	Male New Zealand rabbits subjected to 30 min coronary artery occlusion and 3 h reperfusion	Melatonin, 50 mg/kg intravenous before ischemia	Melatonin did not reduce infarct size compared to control; although melatonin reduced markers of oxidative stress

**Table 2:** Clinical studies evaluating melatonin as a therapeutic strategy against IRI

Reference/Trial name	Clinical setting	Trial description	Treatment	Primary endpoint	Secondary endpoints	Outcomes
Melatonin Adjunct in the acute myocaRdial Infarction treated with Angioplasty (MARIA) [54, 56] [55]	STEMI patients	Unicenter, prospective, randomized, double-blind, placebo-controlled, phase 2 n = 146 Randomized patients were divided into tertiles according to symptoms onset-to-balloon time (n = 41;43;41)	Intravenous administration and an intracoronary bolus of melatonin with a total dose of 11.61 mg	Infarct size expressed as a percentage of left ventricular myocardial mass. Measured by magnetic resonance imaging	Clinical events occurring within the first 90 days: death, sustained ventricular arrhythmias, resuscitation from cardiac arrest, cardiogenic shock, heart failure, major bleedings, stroke, need for revascularization, recurrent ischemia, re-infarctions and rehospitalization	No significant differences were found in the percentage of total necrotic myocardial mass as determined by MRI Melatonin given < 2.5 h after symptom onset could reduce infarct size by approximately 40% as measured by MRI
IMPACT trial [87]	STEMI patients	Multicenter, randomized, double-blinded, placebo-controlled N = 40	Intracoronary or intravenous melatonin (total 50 mg) or placebo (isotonic saline)	Myocardial Salvage Index (MSI) assessed by CMR	Infarct size, microvascular obstruction, left ventricular end-diastolic and end-systolic volumes, and left ventricular ejection fraction hs-TnT and CKMB, inflammation and oxidative stress assessed by advanced oxidation protein products (AOPP), malondialdehyde (MDA) and myeloperoxidase (MPO), and 90-day clinical events (nonsustained ventricular arrhythmias, non-fatal cardiac arrest, cardiogenic shock, coronary revascularization with PCI or coronary artery bypass surgery, major bleeding, reinfarction, stent thrombosis, cardiac and non-cardiac rehospitalizations, and death)	No significant differences in all the parameters measured
[78]	STEMI patients	Randomized N = 40	3 mg of melatonin	hs-TnT and CK-MB		Significant reduction of the level of CK-MB in melatonin group; no difference in the mean hs-TnT
[58]	Patients who were undergoing elective CABG	Unicenter, randomized, double-blinded, placebo-controlled. N = 45	Administration of 10 or 20 mg/day Melatonin capsule from the day five before surgery	Ejection fraction (EF%) was measured using echocardiography. Inflammation, oxidative stress and apoptosis biomarkers		Significant increase in ejection fraction % for melatonin-treated groups. Significant reduction in heart rate, decrease in plasma level of cTnI, IL-1β, iNOS and caspase-3
[35]	Patients who were undergoing elective CABG	Prospective, unicenter, randomized, single-blinded placebo-controlled N = 34	60 mg/day melatonin capsules daily starting 5 days before surgery	Apoptosis, inflammatory and cardiac biomarkers Operative and intensive care unit parameters Clinical outcomes (Heart rate, oxygen saturation and blood pressure) ICU and hospital length of stay Medication adherence and quality of recovery. Laboratory parameters		Melatonin administration reduced with statistical significance the levels of NF-kB, IL-6, TNF-α, and troponin-I. Melatonin decreased diastolic blood pressure 24 h postoperative, reduced intubation time, and improved the quality of recovery
[168]	Patients who were undergoing elective CABG	Unicenter, double-blind, randomized placebo-controlled N = 100	3 mg of melatonin the night before surgery, in the morning, and after surgery at bedtime until the third day after CABG	CK-MB and cardiac troponin-I levels, C-reactive protein; erythrocyte sedimentation rate. Arrhythmia, type, and duration of stay in the ICU or hospital		Melatonin reduced CPK-MB levels on the second and third days after surgery. The duration of ICU hospitalization was shorter
[202]	Patients who were undergoing elective CABG	Randomized, open-label, placebo-controlled. N = 60	5 mg of melatonin starting from 24 h before the operation for 3 times and a single dose 1 h before the operation	Cardiac troponin-I levels	Lactate, MDA, and TNF-α levels	Troponin I, lactate, MDA and TNF-α levels in the melatonin group were significantly lower
[12]	Patients who were undergoing elective CABG	Randomized controlled N = 76	12 mg sublingual melatonin the evening before and 1 h before surgery	Atrial fibrillation. hs-CRP, CK-MB, and cTnT levels		The duration of atrial fibrillation, the levels of hs-CRP and CK-MB, 24 h after surgery were significantly lower
[86]	Patients who were undergoing elective CABG	Randomized, double-blind N = 130	3 mg of melatonin from 3 days before surgery until the day of discharge	Cardiac biomarkers (troponin and CK-MB)		No significant difference in troponin and CK-MB

## Pathophysiology of cardiac IRI

### Clinical significance of IRI

IRI plays a key role in worsening clinical outcomes in cardiovascular diseases, especially during acute MI and cardiac surgery, leading to increased infarct size, impaired ventricular function, and increased risk of heart failure and arrhythmia, thus undermining the benefits of reperfusion therapy [96]. Based on the Global Burden of Disease Study 2021 (GBD 2021), ischemic heart disease (IHD) continues to be the foremost cause of mortality and morbidity globally, accounting for approximately 8.99 million deaths in 2021 [44]. Patients who survive IHD often experience long-term complications, leading to reduced survival and weakened quality of life, partly because of myocardial IRI [44].

MI occurs when a coronary artery is narrowed or obstructed, leading to local ischemia, hypoxia and secondary myocardial damage. When the ischemic insult is caused by complete obstruction of coronary blood flow, patients develop ST-segment elevation myocardial infarction (STEMI), the type of MI most associated with IRI. This represents a clinical emergency, and rapid revascularization is mandatory to resolve ischemia and improve patients’ outcomes [64]. Years of research have defined reperfusion therapies, such as percutaneous coronary intervention (PCI) and coronary artery bypass grafting (CABG), as the primary treatment strategies for MI and chronic coronary syndromes. In STEMI patients, PCI is generally the preferred reperfusion strategy; however, urgent CABG may be indicated in cases of complex coronary anatomy or PCI failure. CABG, in turn, is often the preferred revascularization strategy in patients with chronic ischemia and complex multivessel coronary artery disease [26]. However, according to experimental studies on animal models [242], these interventions can also cause myocardial, vascular, or electrophysiological dysfunction, contributing to up to 50% of the final infarct size, due to IRI. In fact, the gradual adoption of both new and well-established evidence-based treatments for STEMI patients over the past 20 years has been linked to improved survival rates and a reduced risk of recurrent ischemic events. However, progress has plateaued since around 2008 [217]. While acute MI has significantly declined in recent decades due to the success and wider accessibility of rapid reperfusion through PCI, 1-year mortality rates remain between 14.8% and 21.1% according to four European MI registries [17]. Although significant progress has been made in revascularization techniques and perioperative management, no pharmacological therapy has yet been approved specifically to target IRI. This highlights the need for the development of effective cardioprotective strategies. Understanding the mechanisms and developing effective prevention strategies for IRI remains a key objective in lowering the overall burden of cardiovascular disease.

### Mitochondrial impairments during cardiac IRI

To sustain its essential functions, cardiac tissue requires a substantial supply of ATP, with approximately 90% generated via oxidative phosphorylation [165]. Consequently, mitochondria occupy nearly 30% of the cardiomyocyte volume [184]. Most pathological consequences associated with IRI are closely linked to mitochondrial dysfunction, which occurs during both the ischemic and reperfusion phases. During ischemia, the interruption of coronary blood flow leads to acidosis and depletion of oxygen, impairing mitochondrial ATP synthesis via the electron transport chain. This is accompanied by an increase in intracellular Ca^2⁺^ levels, which compromises cell viability. Reperfusion restores oxygen and substrates for ATP production and reestablishes physiological pH, which is essential for tissue recovery [16]. However, in severely damaged cardiomyocytes, this restoration may paradoxically exacerbate injury. Upon reperfusion, intracellular Ca^2⁺^ in excess is buffered by mitochondria, promoting oxidative stress with an accumulation of ROS [186], opening of the mPTP, and activation of apoptotic pathways. mPTP opening triggers a cascade of deleterious effects, including mitochondrial membrane depolarization, mitochondrial swelling, and the release of pro-apoptotic factors such as cytochrome c [178]. Besides these events, during ischemia, the lack of oxygen acts as an energetic stressor and induces the reduction in mitochondrial biogenesis and an increment in the mitophagic process. In addition, the I/R process induces an impairment in the balance of mitochondrial fission and fusion proteins, altering mitochondrial quality control and metabolism [188]. The activation of these multiple mitochondrial pathways during IRI results in the loss of tight regulation of the mitochondrial network, leading to a clearly dysfunctional mitochondrial state [172].

## Mitochondrial modulation by melatonin in cardiac IRI

### The role of melatonin receptors in cardiac IRI

The mechanism of action of melatonin is complex and involves G protein-coupled receptors known as melatonin receptor 1 (MT1, Mel1A, or MTNR1A) and melatonin receptor 2 (MT2, Mel1B, or MTNR1B). MT1 and MT2 show differential expression across various tissues, both being highly expressed in the cardiovascular system, which suggests a crucial role for melatonin in cardiac physiology [63, 83, 90]. In addition to the membrane cell receptors MT1 and MT2 and once entered the cell, melatonin can bind to quinone oxidoreductase 2 (QR2), identified as MT3 receptor. The binding of melatonin to MT3 leads to the reduction in the ROS production [28]. In fact, melatonin is recognized as a natural antioxidant and free-radical scavenger 26501252 [219]. In addition, MT3 has been associated with the ability of melatonin to regulate neutrophil infiltration and to reduce granulocyte migration via endothelium, modulating inflammatory responses [38].

Of note, most of the total amount of melatonin is defined as extrapineal, being synthesized in multiple tissues by mitochondria and acting as a paracrine hormone in controlling metabolism and oxidative stress [191]. Recent evidence revealed the presence of the MT1 receptor in neuronal mitochondria where it mediates ischemic injury through an “automitocrine” mechanism: this receptor is activated by melatonin produced by mitochondria and controls apoptosis activation [214]. In support of these data, a mitochondria-targeted photoactivatable melatonin ligand has been synthesized. This tool localizes and accumulates in the mitochondrial matrix, where it acts on MT1 receptors, inhibiting mitochondrial respiration [208]. Elucidating the mechanisms and temporal dynamics of melatonin synthesis in mitochondria will be crucial to fully appreciate its direct role in modulating mitochondrial function.

Melatonin has demonstrated protective effects against IRI in various organs including heart [243], liver [204] and brain [195, 221]. The mechanisms underlying melatonin’s cardioprotective action involve both its antioxidant properties and the influence on receptor-dependent or independent signaling pathways, even though the contribution of these mechanisms is not yet fully elucidated. Some cardioprotective effects have been primarily explored in studies utilizing luzindole, a non-selective melatonin receptor antagonist [77, 246], suggesting that these effects are largely mediated through the activation of plasma membrane receptors. Notably, recent studies indicate major implications for MT2 receptor compared to MT1. In experiments with MT1- or MT2-silenced mice exposed to IRI, melatonin’s protective effects on myocardial oxidative and nitrative stress, mitochondrial dysfunction, and apoptosis were not observed in MT2-silenced hearts [90]. Similar data were obtained using a specific MT2 blocker (4P-PDOT) which showed that melatonin reduces cardiac IRI through MT2 activation [205]. These findings are consistent with data obtained from ex vivo studies on rat hearts perfused using a Langendorff system and treated with ramelteon, a melatonin receptor agonist. The cardioprotective effects of ramelteon were shown to be dependent on melatonin receptors, particularly MT2, as co-administration of 4P-PDOT abolished ramelteon’s protective effects on infarct size, suggesting a critical role for MT2 activation in cardioprotection against IRI [210, 211].

### Effects on oxidative stress

**Fig. 1:**
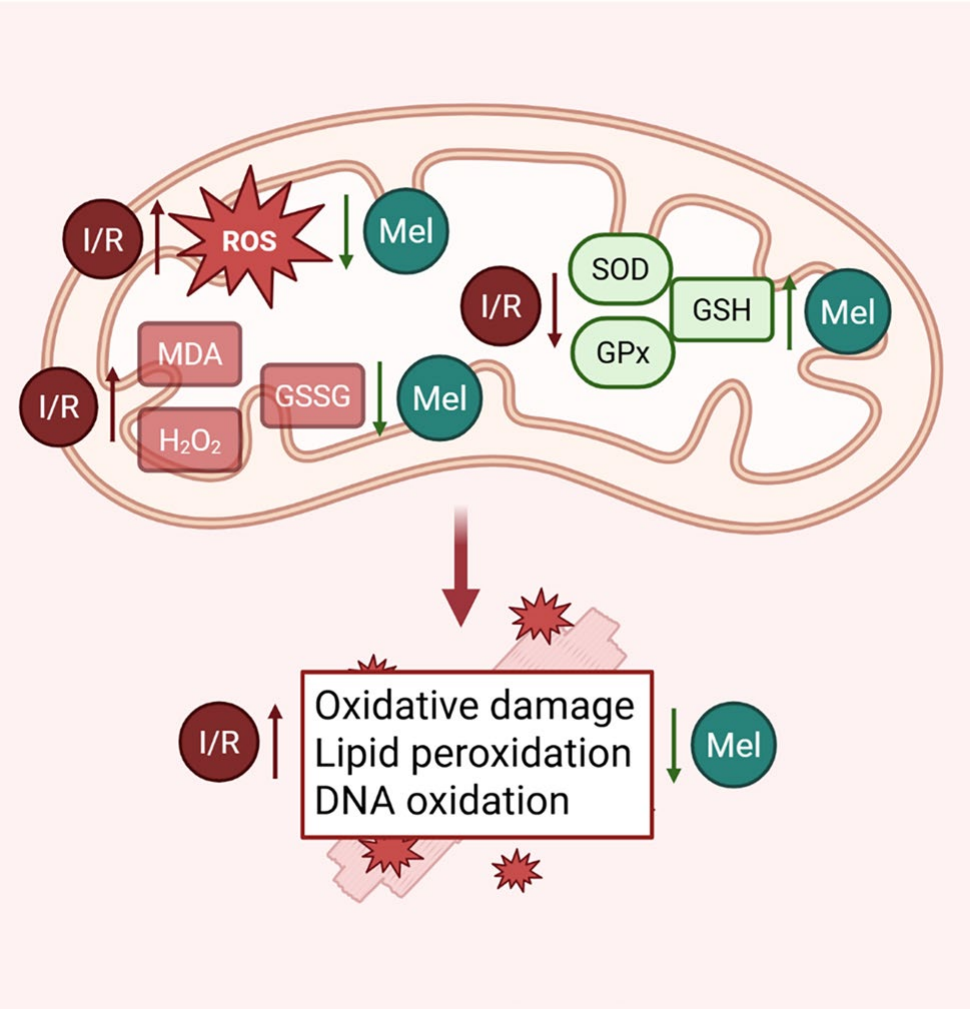
Antioxidant actions of melatonin on mitochondria during IRI. Melatonin exerts potent antioxidant effects at the mitochondrial level by directly scavenging ROS and by enhancing endogenous antioxidant defenses. These actions reduce mitochondrial oxidative damage, limit lipid peroxidation of membranes, mitochondrial DNA oxidation, and dysfunction of the electron transport chain, thereby preserving mitochondrial integrity and cellular viability. Created with BioRender.com

In healthy cells, ROS levels are tightly regulated by a balance between their production and their scavenging by antioxidant defense mechanisms [117]. Following IRI, the sudden burst of ROS results in oxidative damage, compromising cellular structures such as membranes, through lipid peroxidation, and inducing DNA oxidation. Notably, ROS exert a dual role as key signaling molecules and therapeutic targets in cardioprotection, highlighting the need for tightly regulated redox balance to mitigate myocardial IRI [105]. Melatonin has long been recognized for its antioxidant properties, acting both as a free-radical scavenger and by modulating the expression of antioxidant enzymes [185]. These results are also confirmed in animal models of cardiac IRI. In fact, an in vivo study in aged rats demonstrated the cardioprotective effects of melatonin against IRI, primarily through its antioxidant properties [107]. Melatonin reduced mitochondrial ROS production and levels of oxidative stress markers such as malondialdehyde (MDA), while restoring the activities of the enzymatic antioxidants superoxide dismutase (SOD) and glutathione peroxidase (GPx), as well as mitochondrial membrane potential [107]. Similar data were found in experiments conducted on both isolated rat hearts and primary neonatal cardiomyocytes subjected to I/R, which linked melatonin to the Janus kinase 2/signal transducer and activator of transcription 3 (JAK2/STAT3) signaling pathway [239]. This is in line with the emerging role of mitochondrial STAT3 in coordinating survival signaling and preserving mitochondrial function during IRI 37620559 [126]. Melatonin administration was associated with a reduction in markers of mitochondrial oxidative damage, including increased SOD activity and decreased levels of hydrogen peroxide (H₂O₂), MDA, and oxidized glutathione (GSSG) [239]. In addition, experiments involving coronary artery ligation in rats treated intraperitoneally for 10 min before ligation with three different doses of melatonin (2.5, 5 and 10 mg/kg) showed that melatonin improved left ventricular function after ischemia [140]. At the cellular level, melatonin pretreatment also restored ATP synthesis capacity, reduced MDA and Ca^2⁺^ concentrations, and increased glutathione (GSH) levels with the 10 mg/kg dose exhibiting the most pronounced protective effects compared to the lower ones [140]. In a subsequent study, the same dose of melatonin (10 mg/kg) was administered at different time points to rats undergoing left anterior descending artery (LAD) ligation [205]. Melatonin given 15 min before ligation, 15 min after ligation during ischemia, or at the onset of reperfusion consistently led to a reduction in infarct size and improved left ventricular function, regardless of the timing of administration. These cardioprotective effects were associated with improved mitochondrial function, specifically characterized by reduced ROS levels, decreased mitochondrial swelling, and attenuated membrane depolarization (Fig. 1) [205].

Notably, the antioxidant effects of melatonin have been associated with activation of the MT2 receptor. In fact, MT2 expression is selectively increased in ischemic hearts treated with melatonin; concomitantly, in vivo cardiac-specific MT2 silencing by minicircle vectors leads to the loss of melatonin’s positive effects on cardiac injury [90]. MT2 activation by melatonin has been linked to protective effects both on endoplasmic reticulum (ER) and mitochondria, with a reduction of ER stress and apoptosis following I/R and a total reduction of oxidative and nitrative stress [90]. In addition, similar effects of single-dose melatonin treatment against IRI were demonstrated using a prediabetic obese rat model. The study showed that the pretreatment of rats with a 10 mg/kg dose of melatonin attenuated cardiac IRI, improving mitochondrial function, reducing oxidative stress, and enhancing autophagy [206]. Administration of melatonin during ischemia or at the onset of reperfusion at the dose of 20 mg/kg had similar positive effects. Notably, all beneficial effects were abolished by co-administration of 4P-PDOT, a selective MT2 receptor antagonist [206]. In conclusion, the antioxidant-mediated cardioprotective effects of melatonin are well established in preclinical models, underscoring its promising potential as a therapeutic agent.

### Modulation of mPTP opening

mPTP is a multiprotein complex and its precise composition has been studied for several years but is not completely defined. Several data showed the involvement of F_1_/F_O_ ATP synthase in mPTP activity. In addition, adenine nucleotide translocase (ANT) has been proposed as a candidate for the pore-forming protein [20, 120, 161]. mPTP activity is finely regulated by Ca^2+^, Mg^2+^, Sr^2+^, ADP and ATP, P_i_, and fatty acids, in addition to several proteins, such as oligomycin sensitivity conferral protein (OSCP), and cyclophilin D (CypD) [162].

The key role of the mPTP in cardiac reperfusion injury has been widely shown. Its opening leads to mitochondrial depolarization, resulting in cardiomyocyte death via apoptosis or necrosis, mitochondrial swelling, loss of respiratory chain function, and release of pro-apoptotic factors [21]. Several data showed that during ischemia, mPTP opening is inhibited, due to acidic conditions, while, upon reperfusion, the mitochondrial Ca^2+^ entry, the burst of ROS production, and the recovery of pH all lead to mPTP opening in a few minutes [159]. Given the harmful consequence of mPTP opening, the search and development of techniques and/or drugs able to control mPTP opening have become crucial for reducing IRI. To highlight molecular pathways through which this hormone exerts cardioprotection, several research groups have investigated the effect of melatonin on the modulation of the mPTP. In 2022, Bai et al. investigated the transient effects of melatonin in vitro using H9C2 cells subjected to I/R. Their findings demonstrated that the earlier the melatonin was administered following ischemia, the greater its cardioprotective effect was [10]. Concomitantly, melatonin reduced apoptosis by the inhibition of mPTP opening and preservation of mitochondrial membrane potential, and this effect was found to be linked to the suppression of mitophagy [10]. Similar data were obtained using animal models of cardiac IRI. Petrosillo et al. demonstrated in an ex vivo model of I/R that melatonin treatment ameliorates functional parameters of the heart, reducing infarct size and lactate dehydrogenase (LDH) release [181]. Mitochondria isolated from treated hearts were less sensitive to Ca^2+^ and presented a reduced mPTP opening. The observed protective effect was ascribed to melatonin’s capacity to inhibit cardiolipin oxidation, thus preventing cytochrome c release from the mitochondrial membrane [181]. Cardiolipin is a unique phospholipid localized and synthesized in the IMM [174], essential for several mitochondrial functions. Studies carried out by the same authors linked peroxidation of cardiolipin not only to cytochrome c release but also to a lower threshold of Ca^2+^ for mPTP opening induction [180]. Further evidence of melatonin’s modulation of this molecular target comes from a study on isolated mitochondria of aged rats [182]. Melatonin treatment prevented the susceptibility to Ca^2+^-induced activation of mPTP and the release of cytochrome c derived from the aging conditions through the reduction of cardiolipin oxidation [182].

**Fig. 2:**
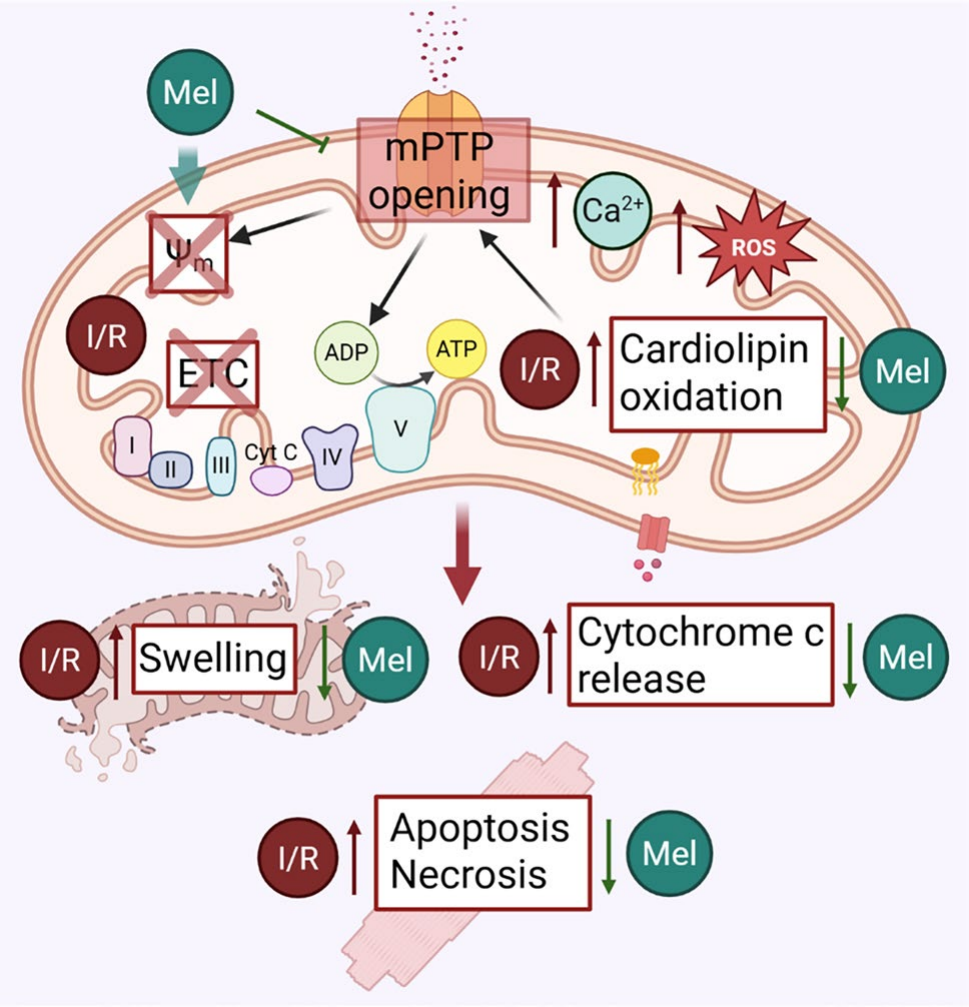
Melatonin-mediated inhibition of mPTP opening during IRI. Melatonin preserving mitochondrial membrane potential and preventing mitochondrial swelling, cytochrome c release, and ATP depletion maintains mitochondrial integrity, limits apoptotic and necrotic cell death, and improves cardiomyocyte survival during reperfusion. Created with BioRender.com

Comparable findings were reported in a study on mice models of cardiac IRI, where melatonin administration preserved cardiac function compared to the control littermates, although distinct molecular pathways were explored in that study. Melatonin reduced endothelial damage and increased survival of endothelial cells of the cardiac microcirculation [253]. The protective effect was exerted both through the inhibition of mPTP opening and the melatonin’s ability to reduce dynamin-related protein 1 (Drp1)-dependent mitochondrial fission, to inhibit hexokinase 2 (HK2) dissociation from mitochondria, to enhance voltage-dependent anion channel (VDAC1) oligomerization and VDAC1–HK2 interaction, and to ultimately decrease PTEN-induced putative kinase 1 (PINK1)/Parkin upregulation and mitophagy induction [253]. A recently published study on mitochondria isolated from porcine aortic endothelial cells showed that melatonin can bind the F_1_/F_O_ ATP synthase in a Ca^2+^-dependent manner, inhibiting mPTP opening and ROS levels following I/R [2]. Comparable to cardioprotective approaches that engage pro-survival signaling cascades that limit reperfusion injury [134], melatonin also confers cardioprotection during reperfusion by activating the RISK pathway (PKB/Akt and ERK1/2) and the SAFE pathway (STAT3), which together preserve mitochondrial function and inhibit mPTP opening [142, 241]. The RISK pathway is in fact activated by various pharmacological agents as a key protective mechanism, leading to a significant reduction in infarct size and enhanced cardiac protection following IRI [36]. Taken together, these findings provide compelling evidence for the role of melatonin in modulating the activity of the mPTP during IRI (Fig. 2). By limiting mPTP opening, melatonin helps to preserve mitochondrial integrity, reduce cell death, and ultimately attenuate myocardial injury, thereby contributing significantly to its cardioprotective effects observed in in vitro and preclinical models (Table 1).

### Impact on mitochondrial homeostasis (biogenesis/dynamics/mitophagy)

**Fig. 3:**
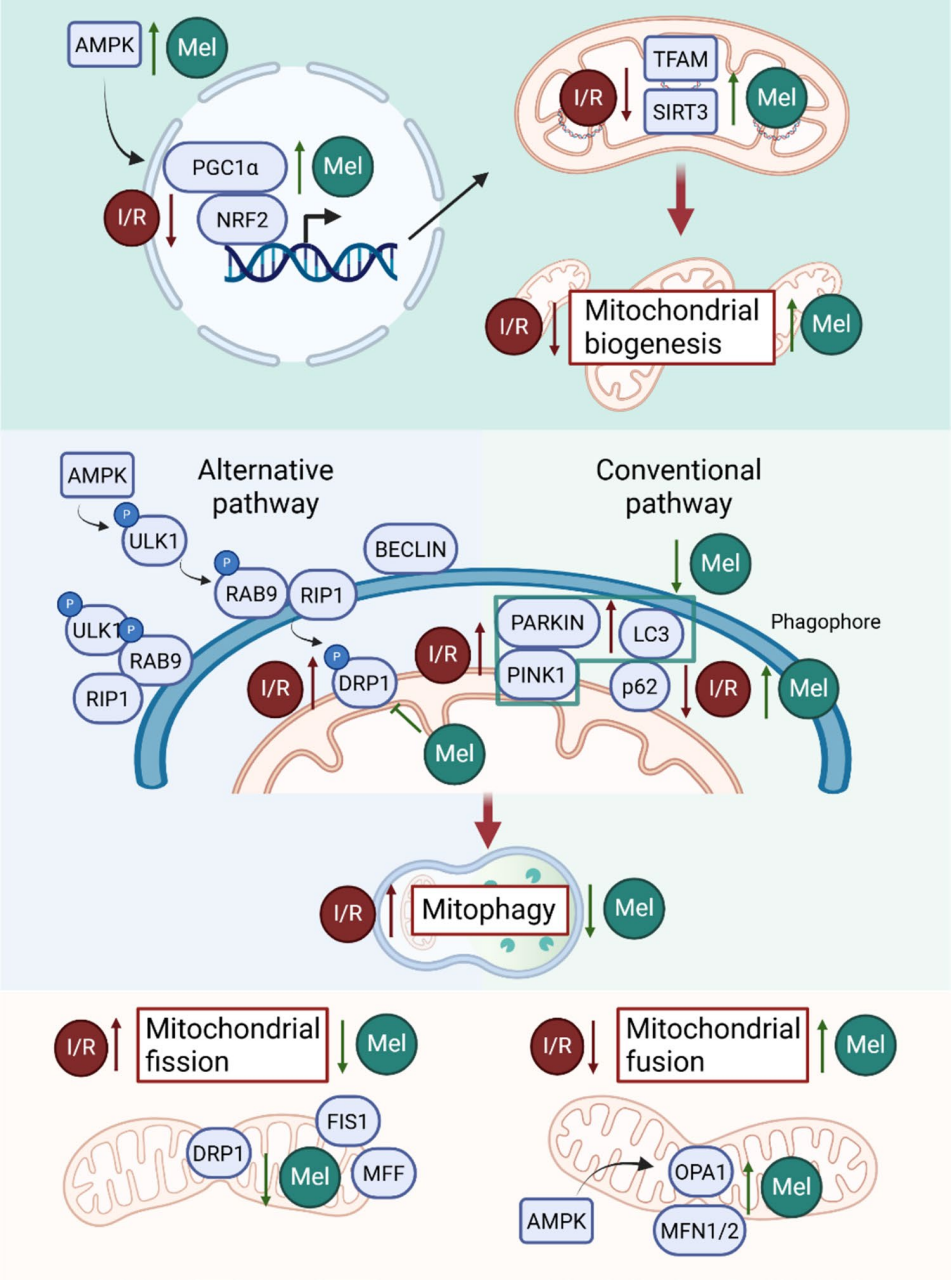
Melatonin regulation of mitochondrial biogenesis, mitophagy, and dynamics (fission/fusion) in IRI. Melatonin supports mitochondrial quality control during IRI by promoting biogenesis, modulating mitophagy, and restoring the balance between mitochondrial fission and fusion. These coordinated actions help preserve mitochondrial integrity and reduce cardiomyocyte injury. Created with BioRender.com

To maintain energy supply, mitochondrial homeostasis is finely regulated by different processes, such as mitochondrial fission and fusion, mitophagy, and biogenesis. All these processes are impaired in cardiac IRI, with a consequent reduction in mitochondrial turnover (Fig. 3).

#### Mitochondrial biogenesis

Mitochondrial biogenesis is the process by which cells increase the number and size of mitochondria in response to elevated energy demands or other signals. This complex process involves the generation of new mitochondria and the expansion of mitochondrial mass, requiring tightly regulated coordination between nuclear and mitochondrial genomes [138]. Accumulating evidence from both in vitro and in vivo studies has shown that melatonin plays a role in regulating mitochondrial biogenesis, likely through its ability to modulate key signaling pathways involved in mitochondrial function, energy metabolism, and cellular homeostasis (Fig. 3). In vitro studies on neonatal mouse ventricular cardiomyocytes demonstrated that melatonin treatment restored the mRNA levels of key biomarkers of mitochondrial biogenesis, which were lowered by I/R: peroxisome proliferator-activated receptor gamma coactivator 1-alpha (PGC1α), Transcription factor A, mitochondrial (TFAM), nuclear factor erythroid 2-related factor 2 (Nrf2), and Sirtuin 3 (SIRT3). This effect is protective against mitochondrial-dependent apoptosis and is mediated through the activation of the 5’ adenosine monophosphate-activated protein kinase (AMPK) pathway [187]. AMPK is a kinase that acts as a cellular energy sensor and regulates cardiomyocyte metabolism, autophagy, apoptosis, and mitochondrial function, including biogenesis, fission/fusion, mitophagy, and mPTP opening [18]. In addition, it has been shown that melatonin is responsible for the restoration of Mitofusin 2 (MFN2) and optic atrophy 1 (OPA1) mRNA levels, and the consequent sustaining of mitochondrial fusion in a PGC1α-dependent manner [187].

Additional evidence relates the protective effect of melatonin against cardiac IRI to the AMPK-PGC1α-SIRT3 signaling pathway, three important mediators of mitochondrial metabolism which regulate both mitochondrial biogenesis and oxidative stress in a type 1 diabetic model subjected to LAD coronary artery ligation [245]. Melatonin has been demonstrated to modulate mitochondrial biogenesis also through its effects on cellular metabolism and redox state. Specifically, it influences mitochondrial acetyl coenzyme A metabolism, alters the activity of respiratory chain complexes, regulates mitochondrial membrane potential, and controls the redox state by acting as a potent antioxidant [132].

#### Mitophagy

Mitophagy is a specialized form of autophagy influenced by ROS, loss of mitochondrial membrane potential and mPTP opening [7] accountable for the degradation and recycling of impaired mitochondria. PINK1/Parkin pathway is one of the most well-studied mechanisms of mitophagy [18], even though recent studies revealed that in ischemic conditions, mitophagy is activated in heart, through the formation of a protein complex consisting of unc-51 like autophagy-activating kinase 1 (ULK1), Rab9, receptor-interacting serine/threonine protein kinase 1 (RIP1) and Drp1 [197]. Hence, mitophagy is a tightly regulated process, and IRI may induce its excessive activation, resulting in impaired mitochondrial homeostasis and increased cell death [222, 225]. Evidence supporting increased activation of mitophagy by IRI derives from an in vitro study on H9C2 cells subjected to I/R. Indeed, under these conditions, H9C2 cells showed an increase in oxidative stress, along with a reduction in cell viability. Melatonin treatment restored the above-mentioned mitochondrial dysfunctions by reducing the expression of Parkin, Beclin1, BCL2-interacting protein 3-like (BNIP3L, also known as NIX) and the LC3-II/LC3-I ratio, while increasing the expression of p62 [237]. Using a combined approach of agonists, antagonists and inhibitors, it was shown that these effects are mediated through the MT2/SIRT3/FOXO3a signaling pathway [237]. An alternative mechanism of mitophagy attenuation following IRI is mediated by the additional contribution of retinoic acid-related orphan receptor alpha (RORα), a nuclear receptor of melatonin. In fact, silencing of RORα or SIRT3 abolished the cardioprotective effect of melatonin [11, 238]. Evidence supporting the role of SIRT3 in melatonin’s mechanism of action also derives from a myocardial I/R rat model, treated with SIRT3 inhibitor (3-TYP) in addition to melatonin [230]. Under this condition, mitophagic markers, oxidative stress and mitochondrial function were evaluated, demonstrating that melatonin treatment can reduce IRI by reducing excessive mitophagy and improving mitochondrial parameters. However, co-administration of the two compounds abolished the protective effects of melatonin [230]. Melatonin’s modulation of mitophagy occurred not only through the conventional pathway but also via the alternative pathway, as shown in a study using isolated rat hearts subjected to the Langendorff perfusion system. Two different concentrations of melatonin (0.3 μM and 50 μM) were shown to upregulate autophagy and to inhibit mitochondrial fission [57]. Specifically, melatonin treatment had a small effect on the PINK1/Parkin pathway and induced a consistent increase in Drp1 phosphorylation at Ser637, which is associated with its inactivation, and altered intracellular levels of ULK1 and Rab9, promoting their cytosolic localization [57]. While most studies have focused on the detrimental effects of I/R on cardiomyocytes in the context of melatonin-mediated protection, Zhou et al. investigated the role of melatonin in modulating platelet activation in a mouse model of IRI. Specifically, melatonin treatment preserved cardiac function and reduced infarct size by attenuating FUN14 domain containing 1 (FUNDC1)-mediated mitophagy and enhancing platelet peroxisome proliferator-activated receptor gamma (PPARγ) expression [251]. Taken together, these findings suggest that melatonin plays a regulatory role in the mitophagy process by fine-tuning the balance between mitochondrial clearance and preservation (Fig. 3). By preventing excessive degradation and turnover of mitochondria, which might otherwise compromise cellular energy homeostasis and contribute to cardiomyocyte injury, melatonin helps maintain mitochondrial integrity, supports optimal bioenergetic function, and ultimately promotes the survival and functional recovery of cardiac cells following IRI.

#### Mitochondrial dynamics

Due to their crucial role in maintaining cellular homeostasis, damaged mitochondria are also controlled through fission, a process regulated by Drp1 and mitochondrial fission protein 1 (FIS1). This process is tightly coordinated with mitochondrial fusion, which is regulated by mitofusin 1 and 2 (MFN1, MFN2) and OPA1. Multiple studies have shown that I/R disrupts the balance between these two mechanisms, with reperfusion specifically leading to increased fission and decreased fusion [99, 149, 254]. The cardioprotective effect of melatonin was shown to be dependent on OPA1 via the AMPK signaling pathway. It activates the AMPK signaling pathway, through modulation of OPA1‐related mitochondrial activity and dynamics, with an anti-apoptotic effect in different cardiovascular models [139]. In addition to promoting mitochondrial fusion, OPA1 plays a central role in shaping cristae within the IMM, thereby influencing mitochondrial metabolism through the assembly of respiratory chain supercomplexes [216]. Cardiac-specific knock-out (KO) mice for OPA1, subjected to I/R, showed that the reduction of the infarction area in melatonin-treated mice was lost. In the same manner, melatonin anti-apoptotic activity linked to mPTP opening inhibition is abolished in OPA1-KO cardiomyocytes, worsening mitochondrial parameters, such as membrane potential and respiratory chain activity [249]. The absence of OPA1 reduces mitophagy after IRI; melatonin, instead, increased LC3-II, Beclin1, and phosphatidylinositol 3-kinase catalytic subunit type 3 (VPS34) expression, promoting mitophagy [249]. Further evidence of impaired mitochondrial dynamics in the context of I/R comes from a study in which, using both in vitro and in vivo models, decreased expression of key mitochondrial fusion regulators, MFN1, MFN2, and OPA1, and increased expression of mitochondrial fission factors, including mitochondrial fission factor (MFF) and Drp1, were observed [146]. Notably, melatonin treatment reversed this dysregulation by restoring mitochondrial function and activating OPA1 via the Yap–Hippo signaling pathway [146]. Mitochondrial fission and fusion processes are closely interconnected with mitophagy and are known to become dysregulated following IRI. Melatonin’s cardioprotective effects have been associated with its ability to restore the balance between these processes, primarily by attenuating excessive mitochondrial fission and promoting mitochondrial fusion (Fig. 3).

## Translational evidence and clinical trials with melatonin

### Large-animal preclinical studies

Building on the encouraging cardioprotective results observed in mice and rat models previously described, it is important to recognize that translation to humans has been limited. This translational gap largely reflects the reliance on preclinical models that do not fully replicate human physiology, as rodents, being nocturnal and having distinct circadian patterns, may respond differently, potentially strengthening the protective effects of melatonin [127]. In fact, when these findings were tested in more predictive and clinically relevant species such as rabbits and especially pigs, the anticipated benefits were substantially weakened or largely neutral (Table 1). A study on porcine models included 20 female pigs randomized to receive either melatonin or saline. Following PCI, animals were given 200 mg of melatonin (or isotonic saline) intravenously and intracoronarily 5 min before reperfusion. Myocardial IRI, evaluated by myocardial salvage index (MSI) and troponin-T levels, showed no significant differences between the melatonin-treated and control groups [62]. Another study on pigs used a closed-chest model subjected to 45 min of ischemia followed by 4 h of reperfusion. Neither oxidative stress markers (MDA) nor inflammatory cytokines (interleukins, IL-1β, IL-6, IL-10) increased significantly after reperfusion in this model, and melatonin did not alter any of these parameters compared with placebo [88]. Similar results were obtained with rabbit models [5, 46]. In anesthetized rabbits subjected to 30 min of coronary occlusion and 3 h of reperfusion, melatonin (10–50 mg/kg, intravenously) had no significant effects on heart rate, blood pressure, or regional myocardial blood flow, and did not reduce infarct size, indicating that while melatonin is safe for the cardiovascular system, it did not confer cardioprotection in this I/R model [46]. These results were confirmed by another study, in which in vivo administration of melatonin at a total dose of 50 mg/kg intravenously in rabbits did not reduce myocardial infarct size during 30 min of ischemia followed by 3 h of reperfusion, despite decreasing lipid peroxidation and modulating SOD activity, indicating that its antioxidant properties alone were insufficient to confer measurable cardioprotection [5].

Despite the disappointing results of melatonin in porcine MI models, other studies in pigs have shown that melatonin can modulate cardiovascular physiology under non-infarct conditions. When infused directly into the coronary circulation, melatonin enhanced coronary blood flow and cardiac performance, effects driven by MT1/MT2 receptor activation, β-adrenergic pathways, and increased nitric oxide (NO) release [83]. These findings indicate that melatonin can exert significant acute hemodynamic and endothelial effects in the normal porcine heart [83]. In pigs subjected to 40 min of coronary occlusion, melatonin did not alter the QT interval or extrasystolic activity but significantly blunted ischemia-induced prolongation of the QRS and Tpeak–Tend intervals, two key markers of conduction slowing and repolarization dispersion. This attenuation was associated with the prevention of early-phase ventricular fibrillation, suggesting that melatonin exerts antiarrhythmic effects in ischemia by limiting detrimental electrical remodeling [15]. Building on these findings, a further porcine study explored melatonin’s electrophysiological actions using detailed intramyocardial recordings during acute MI. Like the prior work, melatonin (4 mg/kg) modulated ischemia-induced electrical disturbances suppressing early-phase ventricular fibrillation [228]. Together, these porcine studies indicate that melatonin can mitigate early arrhythmogenic electrical changes during ischemia in these models, but it is unable to reduce infarct size in MI models [200].

### Clinical trials and meta-analysis

Given the substantial preclinical evidence linking melatonin’s cardioprotective actions to the regulation of mitochondrial homeostasis, this section reviews clinical studies in cardiac IRI (Table 2). These trials did not directly evaluate mitochondrial outcomes, yet they can be interpreted considering the mechanistic insights summarized above. In preclinical studies, melatonin is administered also prior to myocardial I/R to ensure adequate drug concentration at the target site at the onset of reperfusion. However, given the critical importance of reperfusion timing in patients with MI, a cardioprotective agent should be administered immediately at the start of the ischemic process. Thus, treatment strategies acting on reperfusion occurring after ischemia should be administered as early as the ischemic process is diagnosed. However, its favorable safety profile, being an endogenous molecule with good tolerance even at high exogenous doses [4], has made melatonin an appealing candidate for clinical testing.

The MARIA trial investigated the effect of total intravenous administration of a melatonin solution (100 μM; 11.61 mg in 500 ml of sterile saline) in patients with acute MI to assess its cardioprotective potential [54]. This randomized, double-blind, placebo-controlled trial showed that melatonin failed to reduce infarct size, as assessed by cMRI (cardiac magnetic resonance imaging) compared with the control group in patients with STEMI undergoing PCI within 6 h from symptom onset [56]. Nevertheless, the study demonstrated that intracoronary and intravenous administration of melatonin to 73 patients had an acceptable safety and tolerability profile at the used dose (51.7 µmol given 60 min before PCI and an intracoronary bolus of 8.6 µmol given during the procedure) [56]. These findings were inconsistent with previously described in vitro and in vivo evidence supporting the cardioprotective effect of melatonin against IRI. Consequently, the same group performed a post hoc analysis of the MARIA trial, considering the potential influence of ischemia duration and the timing of melatonin administration. To this end, they divided the total sample into tertiles of 41–43 patients based on symptom onset-to-balloon time (136 ± 23 min; 196 ± 19 min; and 249 ± 41 min). Data showed that the effect of melatonin is time dependent. Indeed, administration of melatonin within 2.5 h from the onset of symptoms resulted in the reduction of infarct size by 40%, as assessed by cMRI [55]. However, the limited number of patients in each subgroup may reduce the reliability of the findings and preclude robust statistical conclusions. Even increasing (50 mg administered by intracoronary and intravenous route) or lowering (3 mg) the dosage of melatonin, two randomized clinical trials did not detect an improved myocardial performance in STEMI patients, as assessed by cMRI [55, 61], although melatonin led to a reduction in creatine phosphokinase-MB (CPK-MB) levels compared to the control group [78]. Data emerging from the above-discussed studies have, however, a limited impact due to the relatively low number of patients enrolled, approximately 20 patients per group, which in turn reduces the statistical power and generalizability of the findings. As noted in Ref. [52], further clinical work is required to clarify whether melatonin’s mitochondrial benefits can be harnessed reliably for cardioprotection in patients with IRI, suggesting the need for more rigorous mechanistic and trial design considerations [94].

Many clinical studies investigating the cardioprotective effect of melatonin against IRI use CABG as a model, as this surgical procedure inherently involves a degree of IRI. However, the extent of IRI associated with CABG is considerably lower compared to that occurring during MI. A placebo-controlled study was carried out administering oral melatonin at the dose of 10 and 20 mg/day, starting 5 days prior to surgery [58]. Melatonin treatment increased left ventricular ejection fraction (LVEF) and reduced heart rate compared to placebo, in a dose-independent manner. However, melatonin dose-dependently influenced markers of myocardial injury, such as plasma cardiac troponin-I (cTnI), IL-1β as an inflammatory marker, inducible nitric oxide synthase (iNOS) as an oxidative stress marker, and caspase-3 as an indicator of apoptosis, with lower levels observed in the group receiving the higher dose [58]. A recent clinical trial on 34 patients reported similar findings at a higher melatonin dose (60 mg/day), administered for 5 days prior to surgery. In this study, blood samples were collected at five time points, before treatment initiation and throughout the CABG procedure, to assess plasma levels of apoptotic and inflammatory markers. Melatonin treatment was shown to attenuate IRI associated with CABG by reducing plasma levels of nuclear factor kappa B (NF-κB), IL-6, tumor necrosis factor-alpha (TNF-α), and cTnI [35]. Moreover, the study evaluated clinical outcomes, revealing that melatonin administration led to a reduction in diastolic blood pressure 24 h postoperatively, shortened intubation time, and improved quality of recovery compared to the placebo group [35]. Other pilot studies were carried out using melatonin, by varying doses and timing compared to the above-mentioned reports. The first study is a double-blind, randomized clinical study, and melatonin was administered to 50 patients undergoing CABG the night before surgery, the morning before surgery, and for 3 days postoperatively, at the dose of 3 mg [168]. The second study involved 30 patients who received three doses of 5 mg within the 24 h preceding CABG, and an additional dose 1 h prior to surgery. In both cases, serial measurements of cardiac injury biomarkers during the perioperative period revealed that melatonin treatment significantly reduced levels of cTnI, MDA, lactate, and TNF-α [202] and reduced levels of CPK-MB [168]. In addition, the first study reported that patients experienced a shorter duration of intensive care unit (ICU) hospitalization [168], suggesting a protective effect against CABG-related cardiac injury. Another clinical trial investigating patients undergoing CABG evaluated the efficacy of sublingual melatonin administration at a dose of 12 mg, given the evening before and 1 h prior to surgery [12]. Cardiac biomarkers, including high-sensitivity c-reactive protein (hs-CRP), CPK-MB, and cardiac troponin T (cTnT), were measured. Melatonin treatment resulted in significant reductions in hs-CRP and CPK-MB levels compared to the control group, whereas cTnT levels were not significantly affected [12]. In contrast to previously described studies, a randomized clinical trial involving the same patient population reported no beneficial effects of melatonin treatment on cardiac biomarkers [86]. In this study, 65 out of 130 patients received 3 mg of melatonin daily for 3 days prior to surgery until the day of hospital discharge. Biomarkers were measured 24 h before surgery and at 8 and 24 h postoperatively. The study failed to show a protective effect of melatonin, and this may probably derive from the fact that melatonin dose was not appropriately selected and the study did not have a placebo control group [86].

Over the years, different meta-analyses have collected data from clinical trials using melatonin in the treatment of IRI, with the aim of defining outcomes and offering a comprehensive overview of the current understanding. A recent meta-analysis including six trials comprising a total of 342 patients undergoing CABG demonstrated that melatonin administration significantly reduced cTnI and hs-CRP levels; however, it did not result in a statistically significant reduction in CPK-MB levels [68]. Recently, a systematic review and meta-analysis compared seven randomized controlled trials, three studies on MI treated with PCI and four CABG (described in the text). Data indicate that melatonin-treated patients exhibited higher LVEF and lower circulating troponin levels. However, it is important to note that only two of these studies assessed IRI using cMRI, the gold standard for quantifying myocardial infarct size [53]. Subsequently, Lv et al. conducted a similar meta-analysis, but it included studies using different methodologies, timing, and dose of melatonin, as well as studies involving melatonin analogs [145]. These authors analyzed nine studies involving STEMI (four), IHD planned for CABG (three), acute coronary syndrome (one), and coronary heart disease in diabetic patients (one, article retracted in 2025). Of the nine studies, four reported primary outcome measures, including LVEF and infarct size, while the remaining five focused on secondary outcomes, specifically circulating cardiovascular biomarkers. Pooled analysis of the included studies revealed that melatonin administration failed to significantly improve cardiac function or infarct size. Subgroup analyses, instead, revealed that melatonin conferred cardioprotection only when administered during the early phase of ischemia, either intravenously or via intracoronary injection, resulting in improved LVEF and reduced infarct size. The authors reported several limitations to the studies, such as small sample sizes, variability in melatonin dosing and timing, and insufficient reporting of clinical events, highlighting the need for larger, well-designed trials to clarify melatonin’s cardioprotective effects [145].

The conflicting evidence regarding the efficacy of melatonin in myocardial IRI described by clinical studies involving PCI and CABG may be attributed to the intrinsic differences of these two therapeutic approaches as well as to different target populations. In the context of CABG, the non-negligible role of extracorporeal circulation and its duration must be considered, as it can contribute to IRI not only in the myocardium but also in other tissues. In fact, extracorporeal circulation itself induces a systemic inflammatory response characterized by cytokine release, and is associated with potential systemic microischemic events, hypercoagulability, metabolic stress (including elevated circulating catecholamines), and an increased risk of acute kidney injury [130, 234]. Therefore, PCI and CABG represent two different clinical scenarios, particularly in terms of their systemic impact. The fact that melatonin appears to be effective in the setting of CABG may be due to a systemic rather than to a direct myocardial effect, as suggested by several studies in which troponin levels did not decrease significantly, while inflammatory markers such as hs-CRP and IL-6 did. Ideally, cMRI should also be performed in post-CABG patients; however, the studies conducted so far have measured primarily common inflammatory and vasculo-inflammatory biomarkers (except for troponin), thereby limiting comparability with studies involving PCI. In addition, these trials involve distinct patient populations, often differing in the severity of coronary artery disease and comorbidities, all factors that should also be considered. Most importantly, patients enrolled in PCI studies typically present with acute ischemia, whereas those undergoing CABG usually have more stable coronary artery disease. This underscores the importance of distinguishing the effects of melatonin treatment on acute versus chronic ischemia in future trials.

Ultimately, variations in the timing of melatonin administration, particularly when it is given prior to or following the onset of ischemia, can account for the different results observed. To date, no consensus has been reached on the optimal dose, plasma concentration, timing, or route of administration required to achieve cardioprotection. As discussed in this review, several studies support a dose-dependent effect and suggest that pre-ischemic administration yields more consistent cardioprotective outcomes. Serum melatonin levels are largely determined by dosage, and, like other hormones, must be carefully regulated, as both insufficient and excessive levels can disrupt physiological balance. Interestingly, children with heart failure have been found to exhibit elevated melatonin levels that correlate with disease severity [236]. This is surprising, since melatonin is generally cardioprotective and lower levels are typically observed in cardiovascular disease. The increase in melatonin may reflect a compensatory response to worsening heart failure, although further research is needed to confirm this. These findings highlight the importance of precise dosing, as the effects of elevated melatonin remain uncertain [143] This uncertainty, especially regarding clinical translation, is underscored by a very recent, large-scale clinical study presented at the AHA Scientific Sessions 2025, which found that long-term melatonin use in adults with insomnia was associated with a significantly increased risk of heart failure and mortality [192]. Due to its observational design, this study cannot prove causation, and its findings are further limited by potential misclassification of over-the-counter supplement users, diagnostic coding inconsistencies for hospitalizations, a lack of data on insomnia severity and psychiatric comorbidities, and the not peer-reviewed results of the research abstract.

## Other mitochondria-targeted protective strategies and comparative analysis with melatonin

Cardioprotection encompasses all strategies that prevent or mitigate cardiac injury by targeting different mechanisms and phases of injury but is more specifically defined as interventions that reduce infarct size and limit coronary microvascular obstruction [104]. Over the past decades, mitochondria have emerged as central effectors, and this has led to the development of multiple mitochondria-targeted strategies aimed at reducing IRI [22, 24].

### Pharmacological modulation of mitochondrial pathways

#### Oxidative stress

Given the depletion of endogenous antioxidants in hearts following I/R, therapeutic strategies aimed at restoring redox balance have shown efficacy in mitigating injury. These approaches include supplementing exogenous antioxidants (such as vitamins A, C, and E [203, 250]), administering compounds that activate the redox-sensitive transcription factor Nrf2 [223], and utilizing various antioxidant proteins (e.g., humanin and berberine [49]). The consistent cardioprotection observed across these diverse interventions reinforces the central role of oxidative stress in IRI.

N-Acetylcysteine (NAC) is the most extensively investigated antioxidant in clinical trials for myocardial IRI, with early studies showing reduced myocardial oxidative stress and, in combination with nitroglycerin, smaller infarct size and improved myocardial salvage in STEMI patients [176, 226]. Meta-analyses and small trials suggest that NAC administered before or during reperfusion can improve cardiac injury markers and postoperative recovery, but larger phase III trials have failed to demonstrate consistent cardioprotection, likely due to suboptimal dosing or study design [123, 224]. These mixed results indicate that NAC may be more effective at adequate doses or as an adjunctive therapy rather than as a standalone intervention. Perioperative administration of NAC and melatonin attenuates early reperfusion injury in patients undergoing CABG by reducing oxidative stress and myocardial injury markers [202]. Both agents improved antioxidant capacity and limited biochemical and functional signs of reperfusion damage, supporting the concept that targeting redox imbalance during early reperfusion can confer cardioprotection in the surgical setting [202]. Their combined administration was not evaluated, and no conclusions can be drawn regarding potential additive or synergistic effects in this pathological context. Converging evidence from cerebral I/R studies shows that their co-administration exerts synergistic neuroprotection, characterized by a greater reduction in oxidative stress and lipid peroxidation, preservation of antioxidant defenses, and improved histological and functional outcomes compared with either agent alone, supporting the concept that complementary antioxidant mechanisms may enhance protection in IRI across tissues [9, 195, 207].

#### mPTP opening

Numerous cardioprotective interventions have focused on inhibiting the mPTP, whose sustained opening at reperfusion triggers bioenergetic collapse and cell death. Despite strong mechanistic and preclinical rationale, some pharmacological strategies targeting mitochondrial function as adjuncts to reperfusion in STEMI have not yet demonstrated consistent clinical benefit, underscoring the translational gap and the need for better patient selection, timing, and mechanistically informed trial designs [33].

Cyclosporine A (CsA), a mPTP inhibitor which targets CypD, has demonstrated consistent and significant cardioprotection in preclinical models of IRI [98], but it appears to reduce infarct size inconsistently and with variable efficacy across different experimental species in models of reperfused MI [135], and it failed to improve clinical outcomes in patients with STEMI, as reported in the CIRCUS and CYCLE trials [42, 171]. Similarly, NIM811 and sanglifehrin A are non-immunosuppressive CypD inhibitors that limit mPTP opening at reperfusion, preserving mitochondrial oxidative phosphorylation, reducing infarct size, and improving early post-MI cardiac function in preclinical models [114, 175]. Several preclinical mPTP inhibitors with defined mechanisms have emerged in recent years [162]. These include a highly potent new class of nonpeptidic CypD inhibitors (e.g., C105SR) [124]; CypD-independent small molecules (isoxazole 63, triazole TR002) [6]; cinnamic anilides that boost mitochondrial stress tolerance [66]; and targeted Oligomycin A analogs for the c-ring of ATP synthase [67, 163, 177]. Separately, the dual-purpose probe IR-780 both detects at-risk cardiac tissue and inhibits the mPTP by targeting ATP synthase [41].

Despite extensive research on mPTP inhibition as a cardioprotective strategy, there remains a notable paucity of literature investigating the therapeutic combination of established mPTP inhibitors with melatonin. A study on ex vivo models of MI found that the protective effects of the melatonin receptor agonist ramelteon were lost when the mPTP inhibitor CsA was co-administered before ischemia, indicating a negative pharmacological interaction. However, when CsA was given subsequently at reperfusion, cardioprotection was restored, suggesting that ramelteon exerts its effect through an upstream signaling pathway whose benefits are realized via downstream mPTP inhibition [211]. Unlike direct mPTP inhibitors such as cyclosporine A or ATP synthase-targeting compounds that act on single pore-associated components with limited clinical translation, melatonin converges on mPTP inhibition indirectly through shared upstream interactors, including the attenuation of mitochondrial ROS, preservation of cardiolipin–cytochrome c interactions, modulation of mPTP sensitivity to Ca^2^⁺, and activation of RISK/SAFE signaling pathways. This broader, mitochondria-centered mode of action suggests that melatonin could complement more selective mPTP-targeted agents, providing a mechanistic rationale for combination strategies aimed at stabilizing pore regulation at multiple levels during reperfusion.

Another strategy targeting mitochondria involved KAI-9803, that prevent apoptosis by limiting the accumulation and dephosphorylation of the pro-apoptotic BAD (Bcl-2-associated death promoter) [166]. Despite its proposed role in attenuating apoptosis, clinical trials exploring its effect on STEMI patients (DELTA-MI and PROTECTION-AMI) demonstrated good safety but failed to show consistent cardioprotective efficacy [14, 136]. Similarly, TRO40303, which targets the OMM translocator protein (TSPO), demonstrated limited efficacy in the MITOCARE study [8].

Other strategies have aimed to preserve mitochondrial energetics, such as the mitochondria-targeted peptide Elamipretide (SS-31), which binds cardiolipin and prevents its peroxidation by cytochrome c [215]. This peptide has been shown to maintain cristae structure and improve bioenergetic function after IRI, in addition to controlling mitochondrial dynamics processes [3, 196]. Although Elamipretide has demonstrated to be safe and well tolerated in the EMBRACE STEMI trial, it failed to reduce infarct size or improve clinical outcomes, underscoring the persistent challenge of translating mitoprotective strategies into clinical benefit in reperfused STEMI patients [80]. Both melatonin and SS-31 converge on mitochondrial preservation to confer cardioprotection during I/R, not only by binding cardiolipin and limiting mPTP opening and oxidative stress but also by modulating mitophagy and mitochondrial biogenesis; this overlap suggests that combining these agents, or pairing them with other strategies, could synergistically enhance mitochondrial resilience and improve outcomes in IRI. Melatonin may achieve greater efficacy when integrated into multimodal strategies rather than used as a standalone intervention, particularly in clinical settings characterized by comorbidities and complex pathophysiology [47].

#### Mitochondrial biogenesis

Emerging cardioprotective paradigms increasingly target the fundamental process of mitochondrial biogenesis, seeking to counteract the loss of functional mitochondria and metabolic dysfunction that characterizes IHD. As a peroxisome proliferator-activated receptor alpha (PPARα) agonist, the fibrate bezafibrate promotes mitochondrial biogenesis via PGC1α upregulation, offering a mechanistic link to its observed cardioprotection 30400386 [125]. Clinically, this translates to reduced fibrinogen and major cardiovascular events in acute STEMI patients, with long-term data from the Bezafibrate Infarction Prevention (BIP) study confirming decreased cardiac mortality and non-fatal MI in coronary artery disease [148]. In contrast, within the thiazolidinedione class of PPARγ agonists, outcomes are heterogeneous. Pioglitazone is associated with a reduced risk of stroke and MI [122, 141], whereas rosiglitazone is linked to an increased incidence of adverse cardiovascular events [169], underscoring that shared molecular targets do not guarantee uniform clinical effects. Beyond nuclear receptor activation, agents targeting the cellular energy sensor AMPK also confer protection. The AMP mimetic AICAR activates the AMPK/SIRT1/PGC1α axis, supporting mitochondrial function and promoting cardiac recovery post-ischemia in preclinical models [213, 248]. Similarly, the antidiabetic drug metformin, an AMPK activator, enhances mitochondrial biogenesis and has shown cardioprotective potential in diabetic populations [155, 252]. However, its benefits are context-specific, as evidenced by the GIPS-III trial, where metformin failed to improve left ventricular function in non-diabetic STEMI patients both short and long terms [92]. Melatonin elicits cardioprotection as a pleiotropic upstream modulator, engaging antioxidant systems and metabolic stress-response pathways (AMPK/SIRT) to promote mitochondrial resilience through conditioning, resulting in a favorable safety profile suitable for chronic use. In contrast, synthetic agents such as bezafibrate and AICAR function as high-affinity, targeted activators of specific nodes (PPARα, AMPK) designed for acute intervention, offering greater mechanistic precision but might be accompanied by risks of narrow therapeutic windows and context-dependent efficacy.

Current literature provides limited evidence regarding the therapeutic effects of combining melatonin with pharmacological agents that modulate mitochondrial biogenesis. In a 7-week study of diabetic rats, co-administration of either pioglitazone or rosiglitazone with melatonin significantly improved cardiovascular outcomes by reducing blood pressure, normalizing cardiac injury markers (AST, LDH), and lipid profiles, lowering atherogenic risk, attenuating myocardial histopathological damage, and enhancing cardiac antioxidant defenses (SOD, GSH, catalase), demonstrating a synergistic therapeutic potential for treating diabetes-induced cardiovascular complications [1]. Results from in vivo rat models and in vitro neonatal cardiac microvascular endothelial cells (CMECs) showed that combining melatonin with either AICAR or the mTOR inhibitor rapamycin results in a complete loss of melatonin’s cardioprotective efficacy, as these co-treatments abrogate its ability to reduce infarct size, improve cell vitality, and suppress autophagy [40]. Rapamycin, an mTORC1 inhibitor, promotes cardioprotective mitophagy and reduces infarct size, cardiomyocyte apoptosis, and hypertrophy in models of myocardial injury; its induced cardioprotection against IRI is mediated by the JAK2-STAT3 signaling pathway [45, 70, 72].

While melatonin’s broad conditioning could theoretically complement the acute precision of targeted agents like PPARγ agonists, the antagonistic interaction with AICAR or rapamycin demonstrates that successful combination therapy requires mechanistic compatibility to avoid nullifying beneficial effects. Therefore, a successful dual therapeutic strategy must be predicated on a detailed mechanistic understanding to avoid pathway conflict, ensuring that melatonin’s protective effect is paired only with agents that operate on compatible pathways.

#### Mitophagy

Emerging therapeutic strategies specifically seek to enhance mitophagy, the selective autophagy of mitochondria, to eliminate dysfunctional organelles and prevent their contribution to oxidative stress, apoptosis, and energetic failure in the injured heart [71, 160]. The natural compounds spermidine and trehalose have emerged as promising agents capable of enhancing both non-selective autophagy and mitochondrial-specific mitophagy in cardiomyocytes and in vivo models [60, 199]. Trehalose, a natural disaccharide, attenuates adverse cardiac remodeling and improves heart function following MI in mice model by specifically promoting protective autophagy and enhancing mitochondrial quality control. Its cardioprotective effects are mediated through activation of the transcription factor TFEB, which increases lysosomal and autophagic activity, facilitating the removal of damaged mitochondria and protein aggregates in the stressed myocardium [199]. Similarly, spermidine, a naturally occurring polyamine, promotes autophagy by inhibiting EP300, an endogenous autophagy suppressor. It exerts robust cardioprotective and anti-aging effects by promoting cardiomyocyte autophagy and mitophagy, which, in mice, improve diastolic function, reduce cardiac stiffness via titin phosphorylation, and suppress pathological hypertrophy. Epidemiological and experimental evidence collectively position spermidine as a promising dietary intervention to mitigate cardiovascular aging and heart failure [60]. Evidence from zebrafish embryos demonstrated that while both spermidine and melatonin independently showed cardioprotective effects against insecticide-induced cardiac toxicity, rescuing disrupted gene expression, improving heart morphology, and restoring cardiac function, co-treatment with both compounds did not demonstrate a significant additive or synergistic benefit beyond the effects of either agent alone [137]. These results may be speculatively attributed to a saturation of the beneficial response, overlapping action on convergent cellular pathways (e.g., antioxidant defense or mitophagy), and potential insensitivity of the experimental endpoints to detect more nuanced molecular synergy.

#### Mitochondrial dynamics

A promising avenue in cardioprotection involves therapeutic strategies aimed at modulating mitochondrial dynamics, specifically, the balance of fission and fusion, to preserve mitochondrial network integrity, prevent apoptosis, and enhance cellular resilience against IRI. Preclinical studies show that Drp1 inhibition, most notably with mdivi-1, reduces mitochondrial fragmentation, limits mPTP opening, preserves mitochondrial function, and decreases infarct size, with the greatest benefit observed when treatment is applied before ischemia, although concerns remain regarding its specificity [23, 170]. Newer approaches, including the selective Drp1 inhibitor DRP1i27, the fission-suppressing compound isosteviol sodium, repurposed drugs such as hydralazine, and agents like LS-102 that modulate Drp1 phosphorylation, consistently demonstrate cardioprotection by preserving mitochondrial membrane potential, reducing ROS and apoptosis, and improving cardiac function [23, 39, 116, 193, 212]. In parallel, emerging evidence identifies metabolic regulation of mitochondrial fission, such as inositol-dependent control of AMPK activity, as a novel mechanism linking cellular metabolism to mitochondrial dynamics, collectively highlighting mitochondrial fission as a promising therapeutic target in myocardial IRI [108]. Melatonin and Drp1 inhibitors both mitigate pathological mitochondrial fission during cardiac IRI but through distinct strategies: melatonin acts upstream by rebalancing fusion–fission dynamics, primarily via OPA1 activation, while compounds such as mdivi-1 provide targeted, direct inhibition of Drp1 to prevent fragmentation.

### Systemic and non-pharmacological cardioprotective strategies

In addition to direct pharmacotherapy, cardioprotection might also be achieved through systemic physiological interventions, such as ischemic conditioning and regular exercise, which enhance the heart’s intrinsic resilience by promoting adaptive mitochondrial modifications that improve energy metabolism, quality control, and resistance to ischemic stress.

#### Cardioprotective conditioning maneuvers

Ischemic conditioning encompasses local ischemic preconditioning (IPC), ischemic postconditioning (IPost), and remote ischemic conditioning (RIC), all of which involve brief, nonlethal episodes of ischemia applied locally or at a distance to activate endogenous cardioprotective pathways [100]. These pathways converge on mitochondria, where they modulate intracellular pH, generate controlled ROS signaling, inhibit mPTP opening, and ultimately promote cardiomyocyte survival [74, 95]. IPC induces an early and a delayed phase of protection, preserving mitochondrial respiration, membrane potential, ATP production, and ultrastructure, while preventing cytochrome c release, protein oxidation, and apoptosis through activation of mitochondrial ATP-sensitive K^+^ channels [144, 153]. Similarly, IPost protects the heart by promoting transient acidosis and gradual reoxygenation, activating mitochondrial ATP-sensitive K^+^ and mitochondrial Ca^2+^-activated K^+^ channels, reducing mitochondrial oxidative stress and Ca^2^⁺ overload, and preserving mitochondrial integrity and function during reperfusion [43, 109]. In addition, experimental evidence indicates that ischemic conditioning, like melatonin treatment, restores mitochondrial biogenesis by activating PGC1α-dependent pathways, thereby maintaining mitochondrial structure and function, and contributing to cardioprotection [153]. IPC also induces cardioprotective mitophagy via PINK1/Parkin signaling, promoting Parkin- and p62-dependent clearance of damaged mitochondria, a process that is essential for IPC-mediated infarct limitation [110]. Emerging evidence has further linked the benefits of ischemic conditioning to the regulation of mitochondrial dynamics, particularly through reduced mitochondrial fission and enhanced fusion. RIC has been associated with increased expression of the fusion protein OPA1 and suppression of the fission mediator Drp1, thereby limiting ischemia-induced mitochondrial fragmentation [37]. In addition, RIC preserves mitochondrial respiration, especially Complex I-driven respiration, enhances ATP production, increases calcium retention capacity (thereby reducing mPTP opening), and lowers ROS production in human right atrial mitochondria, alongside improved contractile function in atrial tissue [129]. Experimental studies further suggest that humoral factors released during RIC can directly target cardiomyocyte mitochondria, highlighting a mechanistic link between circulating mediators and mitochondrial protection [76].

Numerous preclinical studies demonstrate that ischemic conditioning interventions reduce infarct size [104], and some of these strategies have shown benefit in small clinical proof-of-concept trials in patients with reperfused acute MI [103]. Both IPC and IPost preserve mitochondrial network integrity by upregulating pro-fusion proteins (MFN1 and MFN2) and downregulating the pro-fission protein Drp1, thereby limiting infarct size and reinforcing mitochondrial homeostasis as a central mechanism of cardioprotection [113]. At the signaling level, ischemic conditioning primarily activates endogenous pro-survival cascades, including the RISK (PI3K–Akt, ERK1/2) and SAFE (JAK2/STAT3) pathways, which converge on mitochondria to inhibit mPTP opening, attenuate Ca^2+^ overload, and preserve mitochondrial membrane potential at reperfusion [100].

Only limited experimental evidence currently supports interactions between melatonin and ischemic conditioning. In a rat model of remote ischemic preconditioning, melatonin modulated the cardioprotective effects of conditioning by regulating inflammatory and apoptotic signaling pathways, resulting in reduced myocardial injury after I/R [84]. In contrast, another study reported neutral interactions, showing that melatonin did not modify the infarct-limiting effects of IPC despite attenuating oxidative stress [5]. Together, these divergent findings underscore the complexity of mitochondrial signaling during ischemic conditioning and caution against assuming a universal synergistic effect between melatonin and conditioning strategies. Given the substantial convergence of melatonin and ischemic conditioning on mitochondrial targets, it is conceivable that melatonin may function as a pharmacological surrogate of conditioning rather than a synergistic adjunct. This mechanistic redundancy could contribute to the inconsistent outcomes observed with combination approaches and warrants further investigation in models specifically designed to dissect additive versus overlapping mitochondrial effects.

#### Exercise as a physiological conditioning strategy

Exercise is a powerful non-pharmacological intervention [34] that induces transient yet meaningful adaptations in cardiac mitochondrial function and metabolism, ultimately increasing myocardial resilience to IRI [73, 111, 121]. Although acute exercise increases mitochondrial ROS production, training reduces ROS generation relative to oxygen consumption, preserves respiratory control during hypoxia–reoxygenation, and improves energetic recovery [232]. Exercise reduces myocardial IRI by limiting early mitochondrial ROS production through activation of mitochondrial eNOS/NO and protein S-nitrosylation pathways, thereby inhibiting mPTP opening, while exercise-related factors such as MG53 further preserve mitochondrial integrity by repairing cardiolipin-rich membranes, reducing oxidative stress and excessive mitophagy [27, 32, 85]. Exercise improves mitochondrial quality control after myocardial IRI by promoting mitochondrial biogenesis, preserving the balance between fission and fusion, and enhancing mitophagy and energy metabolism [220]. Specifically, endurance and aerobic training activate PGC1α-dependent pathways to increase mtDNA replication and transcription, enhance respiratory chain activity, suppress excessive mitochondrial fission (via downregulation of Drp1 and Bax), promote fusion proteins such as OPA1, and ultimately preserve mitochondrial function, reduce infarct size, and support myocardial remodeling [79, 115, 247]. The combination of melatonin and exercise synergistically protects the heart against isoproterenol-induced cardiac injury in rats, enhancing mitochondrial biogenesis and function (via PGC1α activation), reducing oxidative stress, preserving ATP production, and limiting apoptosis and inflammation, thereby improving cardiac contractile performance and attenuating myocardial damage [189]. Overall, melatonin and exercise converge on a common mitochondrial protective network yet operate at different temporal and biological levels: melatonin as an acute mitochondrial-targeted intervention, and exercise as a chronic mitochondrial conditioning strategy. Their overlapping but non-identical mechanisms suggest potential complementarity rather than redundancy, supporting the hypothesis that combining melatonin with exercise-induced mitochondrial adaptations may enhance resistance to cardiac IRI under selected conditions.

## Barriers to clinical translation: methodological gaps, comorbidities, and pharmacological interactions

### Optimizing preclinical evaluation of cardioprotective strategies

Preclinical cardioprotection studies often suffer from publication bias, methodological heterogeneity, and reliance on young, healthy animal models, which limit translational relevance [103]. A major obstacle to translating cardioprotective strategies into clinical practice is the lack of rigorous, standardized preclinical testing, which has led to premature clinical trials of therapies with inconsistent efficacy [69]. To address this, research networks have established practical guidelines [25], the IMPACT criteria [131], and multicenter research platforms in small and large animal models to ensure reproducibility and robust evaluation of novel therapies [194]. For example, preclinical models that incorporate the standard background therapies used in STEMI patients, such as opioid agonist, heparin, and platelet inhibitors, have been developed and might be used to better assess cardioprotective agents [97]. Furthermore, as in clinical trials, preclinical studies should move beyond infarct size as the primary endpoint and incorporate long-term outcomes such as cardiac function, remodeling, and heart failure development [101]. Although preclinical studies strongly support IRI as a therapeutic target, several clinical trials have largely failed to show consistent reductions in infarct size or biochemical markers of myocardial necrosis [103]. Interestingly, some of these trials demonstrated improvements in myocardial salvage by cMRI or better clinical outcomes despite minimal changes in infarct size, highlighting limitations in biochemical endpoints. These findings suggest that focusing solely on infarct size may be insufficient, and effective cardioprotection may require addressing additional targets such as coronary microvascular obstruction and mitochondrial function in non-cardiomyocyte cells (endothelial, fibroblast, and smooth muscle cells) [102, 231]. Consequently, translating mitochondrial or cellular protection into clinical benefit likely demands multi-target strategies combining infarct size reduction with preservation of the microvasculature and broader myocardial cell populations [47, 105, 106].

Large-animal models, particularly pigs, are preferable due to species similarity in cardiac physiology and drug bioavailability compared to rodents [127]. Human in vitro and ex vivo models, such as patient-derived induced pluripotent stem cell, hypoxia/reoxygenation of explanted atrial tissue, and living myocardial slices, provide an essential intermediate platform to test mitochondrial-targeted therapies, as they retain clinically relevant features including patient age, sex, and comorbidities [59, 156, 179]. Permeabilized myocardial tissue has proven to be a reliable alternative to isolated mitochondria for assessing mitochondrial function, accurately detecting I/R-induced respiratory dysfunction in both porcine and human cardiac tissue, and thereby offering a practical translational tool to evaluate mitochondrial injury and cardioprotective strategies in clinically relevant settings [59]. In addition, for clinical trial design, appropriate timing of drug administration and careful patient selection are essential. To identify when mitochondrial therapies should be administered and to confirm mitochondrial engagement, assessing mitochondrial function in patients, through cardiac biopsies, measures of cellular energetics, or circulating biomarkers such as lactate/pyruvate ratios, will be crucial [164].

### Impact of comorbidities, risk factors and comedications

Translation of cardioprotection from preclinical studies to patients with acute MI has been challenging because patients with IHD frequently present with comorbidities such as diabetes, hypertension, and obesity; they are often treated with multiple comedications and are generally older [93, 128]. Type 2 diabetes and obesity further compromise cardiac mitochondrial function and may diminish the efficacy of mitoprotective strategies [31]. Diabetes and dyslipidemia impair cardioprotective interventions by disrupting mitochondrial homeostasis. In diabetes, excessive ROS, lipotoxicity, and impaired autophagy [89] led to accumulation of damaged mitochondria, increased cardiomyocyte apoptosis contributing to heart failure and higher post-infarction mortality. Similarly, dyslipidemia limits cardioprotection by promoting mPTP opening, reducing antioxidant enzyme expression [152], and preventing the efficacy of CsA [235], highlighting that comorbid metabolic conditions significantly compromise mitochondrial-targeted cardioprotective strategies. Furthermore, hypertension-induced cardiac damage is fundamentally driven by mitochondrial dysfunction and disturbances in mitochondrial dynamics [167]. Each of these factors can impair mitochondrial function and potentially negate mitoprotective interventions [69, 172]. Among the nonmodifiable risk factors, aging itself is associated with mitochondrial structural abnormalities and increased ROS production in cardiomyocytes, contributing to ventricular dysfunction and maladaptive remodeling [183]. Moreover, sex-related differences in mitochondrial properties (including mPTP sensitivity, oxidative stress, and respiration) have been documented [229] contributing to distinct responses to IRI and the greater myocardial salvage observed in women with STEMI [154]. When considered jointly, aging alters cardiac mitochondrial homeostasis in a sex-dependent manner, with older female hearts, compared to male hearts, showing reduced SIRT1/SIRT3 expression, diminished mitochondrial antioxidant defense, and a pronounced pro-inflammatory shift, highlighting how aging and sex mutually shape vulnerability to mitochondrial dysfunction and cardiovascular disease [13].

In addition to comorbidities, several comedications are known to modulate or damage mitochondrial function by interfering with the respiratory chain (e.g., causing uncoupling) or by inhibiting critical processes such as oxidative phosphorylation, mitochondrial DNA replication, and ADP/ATP translocation [157, 190, 240]. Both type 2 diabetes and its treatments, such as metformin, sodium/glucose cotransporter 2 (SGLT2) inhibitors, and glucagon-like peptide 1 (GLP-1) receptor agonists, can modulate mitochondrial function, influencing mPTP activity, oxidative stress, and mitophagy, and therefore may act as major confounders in trials of mitoprotective therapies [244]. Statins and other lipid-lowering agents influence mitochondrial function in complex and sometimes opposing ways. Statins can inhibit mitochondrial respiration, increase ROS production, and promote mPTP opening, though effects vary by type: for example, atorvastatin reduces infarct size via Akt-eNOS activation, whereas pravastatin may facilitate mPTP opening [82, 209]. Proprotein convertase subtilisin/kexin type 9 (PCSK9) inhibitors, which lower Drp1 phosphorylation and restore mitochondrial ROS in insulin-resistant, dyslipidemic models, can also reduce infarct size, potentially through enhanced autophagy [50, 133]. Overall, a deeper understanding of how pre-existing mitochondrial dysfunction interacts with mitoprotective therapies is essential to advance personalized cardioprotection in clinical practice.

As previously discussed, to improve relevance and reproducibility, researchers should align animal age with the human disease context and clearly discuss age-related limitations [131]. Melatonin production is strongly age dependent. Serum levels reach their highest between 1 and 3 years of age and subsequently decline, dropping by approximately 80% during adolescence and continuing to fall throughout adulthood [227], a pattern also observed in aging rodents [201]. Consequently, studies evaluating melatonin in young animals must be interpreted with caution, as their endogenous melatonin levels differ markedly from those of older animals.

In addition, melatonin levels and actions are significantly influenced by comorbidities such as diabetes [65] or kidney disease [112], which themselves disrupt circadian rhythms, alter melatonin receptor signaling, and modify metabolic pathways. Since these conditions can change both endogenous melatonin availability and its physiological effects, including antioxidant defense, insulin regulation, and renin–angiotensin activity, careful preclinical evaluation in disease-relevant models is essential before translating melatonin-based cardioprotection into clinical trials [143].

Drug–drug interactions are an important consideration for translating melatonin therapy, as melatonin is primarily metabolized by CYP1A2, and many commonly used medications can inhibit or induce this enzyme, thereby altering melatonin levels [147]. CYP1A2 inhibitors (e.g., fluvoxamine, diazepam, oral contraceptives, 5-methoxypsoralen, and caffeine) raise circulating melatonin, whereas tobacco smoke accelerates its metabolism [173]. Additional effects, such as the potential for melatonin to affect hemostatic function [233], may confound both safety and efficacy assessments, underscoring the need to account for comedications in preclinical and clinical research.

## Conclusion

Melatonin has emerged as a promising therapeutic agent for the treatment of cardiac reperfusion injury. Given the key role of mitochondria in the pathophysiology of cardiac IRI, melatonin offers a particularly convincing approach, as it exerts multiple protective effects on mitochondrial function. These include the attenuation of oxidative stress, the enhancement of mitochondrial quality control mechanisms, and the reduction of mitochondrial-mediated cellular damage. These pleiotropic actions make melatonin a strong candidate for cardioprotection in the setting of cardiac injury. Nonetheless, despite encouraging preclinical evidence in mice, further research is essential to disentangle the therapeutic potential of melatonin since rabbit and porcine preclinical studies reported neutral results [5, 46, 62, 88]. Indeed, additional studies are needed to define the precise dosage, timing, and mode of administration. Large-scale clinical trials will allow to validate these findings and to assess the safety and efficacy of melatonin as an adjunctive therapy in MI and other ischemic events. The previously described clinical trials suggest that both the timing and route of melatonin administration are critical factors in achieving a beneficial effect on IRI. However, these studies address IRI in distinct therapeutic settings, such as PCI and CABG, where the systemic contribution to myocardial injury likely differs, and the duration and impact of the ischemic stimulus at baseline can be different. In addition, the available clinical trials to date are limited by small sample sizes and potentially insufficient statistical power. In addition, inconsistencies across studies may stem from differences in outcome measures; some trials assess biomarker release, while others rely on cardiac imaging to quantify IRI, complicating direct comparisons (Table 2). cMRI is the gold standard for assessing cardiac injury after MI, providing precise quantification of infarct size, myocardial edema, and microvascular obstruction. However, in the context of CABG, performing cMRI is often challenging, making cardiac biomarkers such as troponins and CPK-MB the more practical, though less specific, alternatives for evaluating myocardial injury. Infarct size reduction, while essential, represents only one facet of IRI. A comprehensive cardioprotective assessment should incorporate additional parameters such as coronary microvascular obstruction, mitochondrial function across multiple cardiac and non-cardiac cell types, electrophysiological stability, and long-term structural and functional remodeling [47, 102].

Despite decades of research and compelling preclinical evidence, the translation of cardioprotective agents, including melatonin, into effective clinical therapies remains limited [94]. A central reason for this gap lies in the design of preclinical studies, which often fail to reproduce the complex pathophysiology of human cardiac IRI. Future experimental models must therefore move beyond healthy, young rodent systems and adopt species, conditions, and disease states that more faithfully reflect the human clinical scenario. Comorbidities, including diabetes, hypertension, obesity, and aging, as well as common comedications, are now recognized as major determinants of mitochondrial biology and, consequently, of the efficacy of mitochondrial-targeted therapies [93].

Taken together, these considerations emphasize the need for a more rigorous and clinically attuned preclinical pipeline. A deeper understanding of mitochondrial pathophysiology in realistic disease contexts, coupled with more appropriate experimental models and multidimensional endpoints, will be essential to truly evaluate melatonin’s therapeutic potential.

## Data Availability

No new datasets were generated or analyzed for this review article. All figures are original and were created by the authors using BioRender.
